# Maternal expression of miR-let-7d-3p and miR-451a during gestation influences the neuropsychomotor development of 90 days old babies: "Pregnancy care, healthy baby" study

**DOI:** 10.1016/j.jpsychires.2022.12.021

**Published:** 2023-02

**Authors:** Fernanda Nedel, Camila P. Ferrúa, Cainá C. do Amaral, Geovanna P. Corrêa, Roberta G. Silveira, Jéssica P. Trettim, Gabriela K. da Cunha, Adriana B. Klug, Ana Paula Ardais, Tatiane B. Fogaça, Karen A.T. Pinheiro, Rachel K.S.S. Bast, Gabriele Ghisleni, Luciano D. de M Souza, Mariana B. de Matos, Luciana de A. Quevedo, Ricardo T. Pinheiro

**Affiliations:** a Catholic University of Pelotas, Pelotas, RS, Brazil; b San Francisco de Paula University Hospital – Fetal Medicine Service, Pelotas, RS, Brazil; c University of Rio Grande Foundation (FURG), FAMED, Department of Specialized Surgery, Rio Grande/RS, Brazil; d Graduate Program in Biological Sciences: Biochemistry, Department of Biochemistry, Institute of Basic Health Sciences, Federal University of Rio Grande do Sul. Porto Alegre/RS, Brazil

**Keywords:** miR-let-7d-3p, miR-451a, Infant development, Motor development, Cognitive development, Pregnancy

## Abstract

**Introduction:**

Studies on maternal microRNA expression have emerged to better understand regulatory mechanisms during the gestational period, since microRNA expression has been associated with pregnancy disorders.

**Objectives:**

This study aims to investigate the association between the expression of the maternal microRNAs miR-let-7d-3p and miR-451a during the second gestational trimester and neuropsychomotor development at 90 days of life of infants.

**Methods:**

This is a case-control study nested within a cohort, with the groups being divided into dyads in which pregnant women presented Major Depressive Episode (MDE) (n = 64), these being the cases, and their respective controls (no MDE; n = 64). The Bayley Scale III was used to assess the outcome of child development, and MDE was assessed through the Mini International Neuropsychiatric Interview Plus. The analysis of miR-let-7d-3p and miR-451a was done via serum from the pregnant women, utilizing the qRT-PCR (n = 128).

**Results:**

The results indicated a negative association between expression levels of miR-451a (β −3.3 CI95% −6.4;-0.3) and a positive associated of the miR-let-7d-3p with the cognitive development domain (β 1.7 CI95% 0.1; 3.0), and a positive association between expression of miR-let-7d-3p with motor development of the infants (β 1.6 CI95% 0.3; 2.9).

**Conclusion:**

This is a pioneering study on the topic that indicates a biological interrelationship between the miRNAs miR-let-7d-3p and miR-451a evaluated during the pregnancy and the motor and cognitive domains of infant development at 90 days postpartum.

## Highlights

• The expression of maternal miR-let-7d-3p and miR-451a is associated with child development.
• Negative association between maternal expression of miR-451a and child cognitive development.
• Positive association between maternal expression of miR-let-7d-3p and child cognitive.
• Positive association between maternal expression of miR-let-7d-3p and child motor development.
• There is a biological interrelationship between the evaluated miRNAs and child development.

## Graphical abstract

**Figure:**
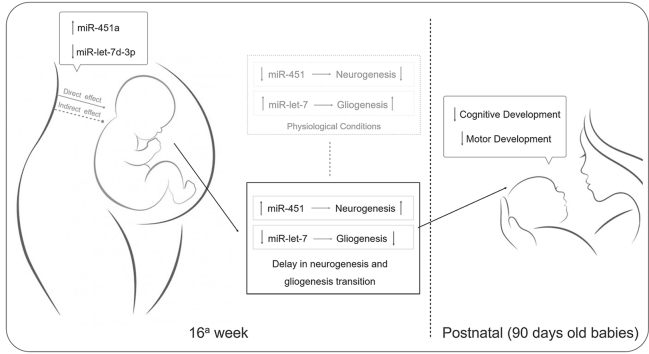

## 1 Introduction

MicroRNAs (miRNAs) are RNA molecules composed of approximately 22 nucleotides, which are involved in the regulation of gene expression. They are present in several tissue and cellular types, regulating biological processes, such as cellular proliferation and differentiation, neurogenesis, angiogenesis and apoptosis (Mohr and Mott, 2015; Ferrúa et al., 2019). Due to this, these small RNA molecules have become a target of interest, as their deregulation may be related to the advent of several pathologies (Huang, 2017; Silva et al., 2018). Moreover, its endogenous expression in peripheral blood, where the expression's profile may considerably modify itself due to pathological conditions, allows one to glimpse at its application in diagnosis, monitoring of evolution, and response to treatment of different pathologies. Thus, miRNAs stemming from peripheral blood have been identified as important biomarkers (Huang, 2017; Dwivedi, 2014).

In the same sense, attention is drawn to the gravidic-puerperal cycle, as this is a moment in a woman's life that encompasses innumerous physical, hormonal, psychological, and social insertion changes (Soma-Pillay et al., 2016; Bjelica et al., 2018). Thus, for a successful pregnancy, the coordination and integration of genes, proteins, and essential nutrients between the maternal-fetal axis is necessary. In this sense, studies on maternal miRNA expression have emerged to better understand regulatory mechanisms during the gestational period, since miRNA expression has been associated with several pregnancy disorders, like recurring abortion (Hosseini et al., 2018; Jairajpuri et al., 2021), preeclampsia (Lv et al., 2019), and premature birth (Cook et al., 2019). These studies contextualize the importance of miRNAs in different outcomes of pregnancy, evidentiating the potential of these small molecules (Hosseini et al., 2018; Jairajpuri et al., 2021; Lv et al., 2019; Cook et al., 2019).

However, miRNAs do not only impact pregnancy outcomes. Increasingly compelling evidence has been suggesting that fetus exposure to adverse situations in the womb influences different outcomes in the offspring, which may be mediated by epigenetic mechanisms, which strongly include miRNAs. Indeed, studies have shown that maternal environmental and physiological factors may directly affect miRNA expression in the baby (Babenko et al., 2015; Su et al., 2020). Moreover, maternal miRNA inherited by the fetus have shown themselves to be important for fetal development, and the deregulation of this expression may lead to important fetal changes, including neurodevelopment changes (Babenko et al., 2015). This is feasible to imagine, as miRNAs are abundantly present in the central nervous system (CNS) and present specific expression patterns on all main cellular types during development, where many of them are emerging as potential regulators of CNS development and its homeostasis (Petri et al., 2014; Cho et al., 2019).

It is highly known that the brain development during the prenatal period, and its improvement during the postnatal period, is highly orchestrated and chronological. It involves periods of neurogenesis, gliogenesis, synaptogenesis, and myelination (Semple et al., 2013). Nowadays, it is understood that neurogenesis, a process of formation, migration, and differentiation of new neurons, precedes gliogenesis, the formation of the glial cell population. The adequate neurogenesis and opportune change from the development program of progenitor domains to gliogenesis are essential to the formation of neural circuits and proper brain function. This occurs mostly around the end of the first trimester of gestation and beginning of the second trimester of pregnancy, where disturbances in these mechanisms may lead to alteration in the child neurodevelopment (Semple et al., 2013; Jiang and Nardelli, 2016; Nakafuku and Del Águila Á, 2020). MiRNAs are strong candidates to participate in the neurogenesis and gliogenesis processes, as well as to mediate the change from the neurogenesis focus to gliogenesis at the opportune moment for adequate development of the CNS(19–21). Indeed, miRNAs have been identified as participants in these mechanisms, where the miR-let-7d-3p (Patterson et al., 2014) and miR-451a (Trattnig et al., 2018) have shown the potential to play a relevant role in this scenario.

Thus it is widely known that a child's nervous system is undergoing constant transformation, and the combination of genetic, epigenetic, and environmental factors is what determines its development (Hochberg et al., 2011). The concept of child development is broad and includes growth, maturation, learning, in addition to psychological and social aspects of the child, where its monitoring aims to promote, protect, and, mainly, detect changes early (de Souza and Veríssimo, 2015). As such, child neurodevelopment deficiencies present themselves as a delay in the acquisition of normal development, being characterized by the non-acquisition of development milestones, according to the sequence of predetermined stages (Shevell, 2010). This delay in development may be associated with several childhood conditions, including conception, pregnancy, and birth (Dornelas et al., 2015).

In view of the above, it is believed that maternal miRNAs, during the gestational period, could serve as an biomarker for the development of motor, cognitive, and language domains during early childhood. However, thus far, there are no studies that have done this assessment. Given this, in this study we investigated the association between the expression of the maternal miRNAs miR-let-7d-3p and miR-451a during the second gestational trimester and neuropsychomotor development at 90 days of life of infants.

## 2 Material and methods

### 2.1 Design

This is a case-control study nested within a population-based cohort study, conducted within a city in the south of Brazil. The cohort project to which this case is linked was approved by the committee of research ethics of the Catholic University of Pelotas, under report number 1.729.653. For more details on sample capturing, read the publications of Pinheiro et al., 2021, 2022.

The groups were divided into dyads (mother-infant) whose mothers had a severe Major Depressive Episode (MDE) during the first two trimesters of pregnancy (cases) and underwent intervention by cognitive behavioral therapy (exposure) (n = 64), and their respective controls (mother-infant dyads) without MDE (n = 64), with these dyads being paired by maternal variables during pregnancy: maternal age in years, schooling in completed years of study, gestational weeks, and socioeconomic level by the classification of the Brazilian Association of Research Companies (ABEP).

### 2.2 Instruments

The Bayley Scale of Infant and Toddler Development III (Bayley-III) was used to assess the outcome of child development at 90 days post-birth through the composite scores of each subscale (cognitive, language, and motor) (Bao et al., 2020). MDE was evaluated with the “A” module of the Mini International Neuropsychiatric Interview (Mini Plus 5.0.0 Brazilian Version). The economic assessment of participants was done through the ABEP classification, with levels being categorized in the following manner: A + B (high levels), C (average levels) and D + E (low levels) (www.abep.org) (Adams and Walls, 2020).

Maternal variables, like age and schooling (collected in completed years and later categorized in terciles), gestational age in weeks, first pregnancy (yes/no), and planned pregnancy (yes/no), were collected via questions of the structured general questionnaire. The Body Mass Index (BMI) was evaluated by weight/height^2^ (kg/m^2^), with weight being measured with the aid of an anthropometric scale and height with a stadiometer. The variables of prematurity and weight of the infant at birth were collected from the infant's medical records, and questions about breastfeeding were responded by the mother. Finally, the variable of “intervention for gestational depression (yes/no)” was inserted in the analysis for control purposes.

### 2.3 Blood sample collection and processing

Blood samples were obtained by venipuncture (10 mL) from all pregnant women (n = 128). The serum blood was immediately centrifuged at 3000 g for 10 min at 4 °C and the supernatant was transferred to RNase/DNase-free tubes and stored at −80 °C until RNA extraction.

### 2.4 RNA extraction and qRT-PCR analysis

Total RNA was extracted from serum using a mirVana PARIS kit (Thermo Fisher Scientific) according to the manufacturer's protocol.

The RNA was reverse transcribed to cDNA using TaqMan miRNA Assays (Thermo Fisher Scientific) according to the manufacturer's protocol. Quantitative PCR was performed using a TaqMan Fast Advanced Master Mix kit (Thermo Fisher Scientific). Thermal cycling was conducted according to the manufacturer's recommended program, and all experiments were performed in duplicate. The TaqMan miRNA Assays used in this study and their Taqman assay IDs are as follows: miR-let-7d-3p (#477848_mir) and miR-451a (#478107_mir). The CT values were normalized according to the delta CT method with the endogenous controls (miR-17–5p). To determine the corresponding ΔCT value, the CT value of the target gene miRNAs was subtracted from the miR-17–5p CT value.

### 2.5 Statistical analysis

The data analysis to investigate the association of gestational levels of miR-let-7d-3p and miR-451a with the outcome of child neuropsychomotor performance at 90 days was done with raw linear regression and later adjusted for: maternal age, gestational weeks, degree of placental maturity, primigestation, planned pregnancy, socioeconomic level, maternal schooling, maternal gestational BMI, gestational depression at the moment of capture (cases and controls), and variables related to obstetric conditions, such as type of birth, prematurity, weight of infant at birth, baby sex in addition to breastfeeding and postpartum depression. The level of significance considered was 0.05.

During further refinement of the adjusted analysis, two models were proposed: the first one with both miRNAs (miR-let-7d-3p and miR-451a) present and autoregulating, and a second one with the input of the expression values of only one of the miRNAs, removing the autoregulation of the other one from the analysis.

## 3 Results

The data analyzed were from 64 dyads (mother-infant) of the group of depressed pregnant women (intervention) and 64 dyads of the group of control pregnant women (non-depressed and without intervention), duly paired and with assessment of the infants 90 days postpartum, totaling a sample N of 128 dyads. The sample characteristics can be seen in Table 1.

**Table 1:** Sample characterization.

	N/mean^a^	%/sd^a^
Sociodemographic variables		
Maternal age		
Up to 23 years	18	14.1
Between 24 and 29 years	61	47.7
30 years or more	49	38.3
**Socioeconomic level**^**a**^		
Higher levels (A/B)	28	22.2
Middle level (C)	81	64.3
Lower levels (D/E)	17	13.5
**Maternal schoolling (in years)**^**a**^	10.7	3.3
**Gestational and perinatal variables**		
**Gestational weeks**^**a**^	16.2	5.6
**Multiparous**		
No	47	36.7
Yes	81	63.3
**Maternal gestation BMI (body mass index)**^**a**^	28.3	6.0
**Prematurity**^**a**^		
No	114	89.8
Yes	13	10.2
**Type of birth**^**a**^		
Vaginal	61	48.0
Cesarean	66	52.0
**Planned pregnancy**		
Yes	72	56.3
No	56	43.8
**Weight of infant at birth (in kg)**^**a**^	3.3	0.5
**Degree of placental maturity**^**a**^		
I	84	70.0
II	29	24.2
III	07	5.8
**Baby sex**		
Male	60	46.9
Female	68	53.1
**Postpartum depression**		
No	114	89.1
Yes	14	10.9
**Breastfeeding**		
No	28	19.4
Yes	100	80.6
**Antenatal Major Depressive Episode (MDE)**		
**MDE**		
No (control)	64	50.0
Yes (exposed)	64	50.0
**TOTAL**	**128**	**100.0**

Continuous data, presented as mean and standard deviation.

Variables with missing.

**Fig. 1:**
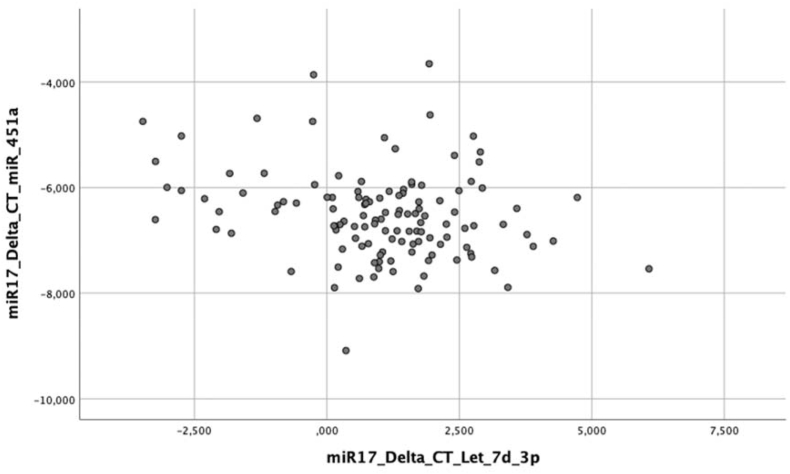
Scatter plot with miRNAs miR-let-7d-3p and miR-451a.

The general average of miR-let-7d-3p was 1.0 (sd ± 1.7) and of miR-451a was −6.5 (sd ± 0.8). A negative (R = −0,204) and statistically significant (p = 0,024) correlation between the microRNAs is observed, indicating that when the expression of one increases, the other decreases and vice versa (Fig. 1: Scatter plot of miRNAs miR-let-7d-3p and miR-451a). The diagnoses of collinearities demonstrate tolerance >0.1 and VIF <10, not indicating multicollinearity between any of the variables tested. Regarding the pairing variables, its effectiveness was verified, with the means of miR-451a and miR-let-7d-3p being similar between the case and control groups (p > 0.05). The mean gestational age within the sample was 16.2 weeks (sd ± 5.6), the most prevalent socioeconomic level was C (64.3%), followed by A + B (22.2%) and D + E (13.5%). Mean maternal age was 28.0 years (sd ± 4.7) and maternal schooling was 10.6 completed years of study (sd ± 3.3).

The linear regression for the outcome of child neurocognitive development shows the association between miR-let-7d-3p and the domain of motor development (β 1.6 CI95% 0.3; 2.9). By analyzing the regression model when inputting the miRNAs one by one, in other words, not adjusting miR-let-7d-3p for miR-451a and vice versa, miR-let-7d-3p also presented association with the cognitive domain (β 1.7 CI95% 0.1; 3.0), as did miR-451a (β −3.3 CI95% −6.4;-0.3). Table 2.

**Table 2:** Expression of maternal gestational miRNAs according to the domains of child development at 90 days postpartum.

	Crude analysis						Adjusted analysis					
	Cognitive development		Language development		Motor development		Cognitive development		Language development		Motor development	
	β	p-value	β	p-value	β	p-value	β	p-value	β	p-value	β	p-value
**miR-let-7d-3p**	1.7 (0.2; 3.2)	0.026	0.5 (−0.3; 1.3)	0.257	2.0 (0.7; 3.3)	0.003	1.4 (−0.1; 3.0)	0.074	0.7 (−0.2; 1.6)	0.118	1.6 (0.3; 2.9)	0.015
**miR-451a**	−3.2 (−6.2;-0.2)	0.035	0.6 (−2.2; 1.1)	0.508	−2.5 (−5.2; 0.2)	0.072	−2.8 (−5.8; 0.2)	0.071	−0.1 (−2.0; 1.9)	0.954	−0.9 (−3.4; 1.6)	0.478
**miR-let-7d-3p**^a^	–	–	–	–	–	–	1.7 (0.1; 3.3)	0.037	0.8 (−0.1; 1.7)	0.094	1.9 (0.7; 3.2)	0.003
**miR-451a**^a^	–	–	–	–	–	–	−3.3 (−6.4;-0.3)	0.031	−3.3 (−2.2; 1.5)	0.720	−1.1 (−3.6; 1.3)	0.372

a Inserted separately in the model.

## 4 Discussion

The present study showed that miR-let-7d-3p and miR-451a expression in the peripheral blood of pregnant women during the first/second trimester of pregnancy is associated with infant development at 90 days of life. Thus, miR-let-7d-3p had a positive association with motor development, and miR-451a and miR-let-7d-3p showed, respectively, a negative and positive association when input separately in the analysis model, highlighting, in the latter one, a possibility of biological interrelation between both miRNAs regarding development of the cognitive domains. It is important to highlight that these results are independent of mothers presenting severe depression during the first two trimesters of pregnancy and undergoing intervention by cognitive behavioral therapy. However, the intervention in this group, proposed by the project, was able to impact on the better motor development of children, as shown in the article published by the group (Pinheiro et al., 2022). The results presented in our article, so far, have not been demonstrated in any study, especially concerning populational studies.

The association found between the expression of maternal miR-let-7d-3p and miR-451a on psychomotor development of infants is likely to be established, as studies have shown that non-coding RNAs from the mother (including miRNAs), both exogenous (from feeding, for instance) as well as endogenous (miRNAs coded from maternal DNA, for instance), can be transferred to the fetus via the placenta and influence fetal development (Su et al., 2020; Li et al., 2015). In this way, it is possible to hypothesize that miR-ler-7d-3p and miR-451a might have a direct action (passed down from the mother to the fetus via the placenta) influencing the baby's neurodevelopment. Thus, impacting the results obtained concerning cognitive and motor domains. However, it is widely known that miRNAs regulate different miRNAs in an orchestrated and complex manner, where a single miRNA may regulate several miRNAs, therefore modulating diverse signaling pathways (Ferrúa et al., 2019). As such, expression of miR-let-7d-3p and miR-451a during the first/second trimester of pregnancy might indirectly influence child neurodevelopment, as its expression might affect signaling cascades, which would culminate in the effects observed on infant development at 90 days of life. (Graphical abstract).

Moreover, the impact of miRNA expression during pregnancy (first/second trimester of pregnancy) on motor and cognitive development at 90 days of life seems to be consistent when taking into consideration that the brain's central systems are established before birth, when global connection are formed, which are indispensable for neural processing in the newborn's brain. Within this spectrum, the first systems to mature are the motor and sensory ones, which support the baby's capacity to interact and receive external information. This relation can be better understood when considering that several regions of the fetus's brain have connection patterns that relate with the baby's subsequent motor capacity, and that the regions which support motor function present higher connectivity with the motor network of babies that developed motor skills faster during childhood (Thomason et al., 2018).

In this scenario, it has been established that the control realized by the miRNAs is important in basically all aspects of nervous system development (Cho et al., 2019). Depending on its target, a miRNA may promote or inhibit the development process. Since the target spectrum for a determined miRNA may change in function of time and space, the miRNA's activity is, many times, specific to a certain context. This is well exemplified by the discoveries that the same miRNAs can be involved in different decisions of cellular fate (for instance, neuron versus glia) or of development stages (for example, localization of axon pathways versus synapse formation) (Rajman and Schratt, 2017). However, generally, miRNAs have demonstrated to be capable of performing important roles in neurogenesis, cellular migration, neuronal maturation, dendritic afforestation, axonal regeneration, and, mainly, synaptic development and brain plasticity (Cho et al., 2019; Rajman and Schratt, 2017). In this context, the expression of maternal miRNAs during pregnancy might impact on the baby's motor and cognitive development, as the systems and circuits of motor and cognitive function develop throughout pregnancy, being widely modulated by miRNAs. Therefore, corroborating with what was exposed above, the higher the connection standard between regions that support motor function and network function of the fetus, the faster motor skills would develop during childhood. Thus, miRNA let-7d-3p, which showed association with infant motor development, could serve as a biomarker capable of guiding approaches during early childhood, which may potentialize the child's capacity, possibly having repercussions in the adult phase.

Regarding the results referring to miR-let-7d-3p positive association with the motor domain, and miR-451a and miR-let-7d-3p showing, respectively, a negative and positive association (input separately in the analysis model), with the infant's cognitive development, it is important to first understand some aspects of neurodevelopment, as well as specificities regarding the evaluated miRNAs. In this sense, as previously mentioned, the first months of the baby's life represent the interregnum in which children acquire their sensory-motor capacities, as can be exemplified by sensitivity to colors, contrasts, and space when it comes to the visual domain, and the ability to elevate the head, roll, press, and sit when it comes to the motor domain (Adolph and Franchak, 2017). Indeed, a recent study demonstrated that infants, during their first six months of life, present a cortex with deep growth of the tissue microstructure in the sensorimotor cortices. When comparing the transcriptomic gene expression between prenatal and postnatal brain tissues, it was revealed that synaptic and myelination processes are dominant contributors to the microstructure of growing tissue in the postnatal stage (Natu et al., 2021).

Moreover, studies have shown that the human brain is in constant expansion during the first postnatal year. C ertain aspects are crucial so that it is possible to decipher which prenatal events are linked to the brain areas that participate in the motor and cognitive domains, especially in relation to the prefrontal cortex. The prefrontal cortex presents a delay in dendritic arborization in comparison with other brain regions, reduction of energy metabolism at 3 months of age, and incomplete myelination by 12 months of age (Sanai et al., 2011; Gilmore et al., 2018). Still regarding myelination, it is necessary to note that it presents a direct age-dependent relationship with general cognitive abilities from three months to five years of age. This may represent a fundamental aspect toward determining the differences in cognitive domain scores between the children analyzed by the study (Deoni et al., 2016).

As such, brain development during the prenatal period associated with its improvement during the postnatal period directly impacts the baby's development of abilities. This process is highly orchestrated and chronological, involving very specific periods of neurogenesis, gliogenesis, synaptogenesis, and myelination (Semple et al., 2013)^.^ T hese being the most specifically elapsed ones within the period evaluated by this study. Nowadays, it is understood that neurogenesis, precedes gliogenesis, which is the result of a mechanism that is inherent and carefully regulated by complex interactions between intrinsic factors (such as epigenetic gene modifications) and extrinsic factors (factors secreted or mediated by contact). Thus, adequate neurogenesis and opportune change from the development program of progenitor domains to gliogenesis are essential to the formation of neural circuits and proper brain function. Disturbances in any of these mechanisms may lead to disorganization and, eventually, dysfunction of the CNS (Semple et al., 2013; Jiang and Nardelli, 2016; Nakafuku and Del Águila Á, 2020).

M iRNAs are strong candidates to participate in the neurogenesis and gliogenesis processes, as well as to mediate the change from the neurogenesis focus to gliogenesis at the opportune moment for adequate development of the CNS. Indeed, miRNAs have been studied and identified as participants of these scenarios, but more specifically, miR-let-7d-3 p (Patterson et al., 2014) and miR-451a (Trattnig et al., 2018) have been showing a relevant role in this context. Patterson et al. (2014), when studying neural progenitor cells originated from human tissue of fetuses at 16 weeks of pregnancy (compatible with the period analyzed in the present study, which was 16.2 weeks [sd ± 5.6]), observed that members of the miR-let-7 family, in which miR-let-7d is found, represent the miRNAs most abundantly expressed at such gestational moment, and which, in contrast, would have intermediate expression in the interregnum between the sixth and seventh weeks. In addition, from the 12th to 19th weeks of pregnancy, it was observed that neural progenitor cells from both the brain and spinal cord presented a differentiation percentage of 70% into glial cells, while less than 20% differentiated into neurons. This could indicate that, at such periods, a decrease in neurogenesis begins, followed by an increase in gliogenesis (Patterson et al., 2014).

In this manner, it appears that during fetal neurodevelopment at the end of the first trimester and beginning of second trimester of pregnancy, more specifically in the 16th week of pregnancy, the miRNAs of the miR-let-7 family present a fundamental role in the determination of cellular fate, turning cell differentiation from neurogenesis to gliogenesis. Additionally, studies with pregnant mice reported that miRNAs from the miR-let-7 family suffer an expression increase in the fetal brain as the pregnancy progresses, and it remains like that until the moment of birth. In a specific analysis of miR-let-7d, it was possible to observe increase of expression levels in progenitors of the ventricular zone of the neocortex and cerebellum of embryos of mice at day 13.5 (Fairchild et al., 2019), which is similar to the pregnancy period that the participants of the present study found themselves in, as both of them correspond to the moment in which the cortical preplate is subdivided into marginal zone and subplate (Prayer et al., 2006).

Bringing this knowledge into the scope of the present study's results, where the reduction of miR-let-7d-3p expression in the serum of pregnant women during the first/second gestational trimester was associated with worse scores in cognitive and motor domains of babies evaluated at 90 days of birth, it is possible to hypothesize that the transition between the neurogenesis and gliogenesis processes may have been harmed and/or delayed due to reduction in the expression of maternal miR-let-7d-3p. As such, this may have impacted in an inadequate formation of synaptic and myelination processes that occur at the period of three months of age, when the infant's brain is still undergoing constant neurodevelopment with maturation of the sensory and motor systems.

The miR-451a, on the other hand, has the increase in its expression levels related to an acceleration of neuronal differentiation, also presenting association with the growth of neuron and dendrite projections, as well as with the formation of the neural network when analyzed in *in vitro* study. Likewise, in an analysis with adult mice, it was observed that miR-451a knockout promotes increased cell proliferative state in neurogenic areas with production of few cells that successfully reach the expected neurogenic fate in the days after proliferation, evidentiating disorders in the differentiation of new cells of neurogenic fate and also in migration (Trattnig et al., 2018).

Therefore, associating this knowledge with the present study's results, it can be hypothesized that pregnant women who presented increase in miR-451a expression levels may have caused increased expression levels of these miRNAs in the fetal brain, causing higher permanence of cellular fate turned toward neurogenesis, implying in a delayed transition to gliogenesis. Thus, resulting in a delay on the postnatal moment of synaptic processes and myelination, generating lower scores for cognitive domains due to the delay in sensory system maturation. Furthermore, the aforementioned hypotheses can also help to explain why there is no statistically significant or sustained difference when one of the miRNAs is adjusted for the other. W hile miR-451a appears to have a higher expression prevalence in the occurrence of neurogenesis and, therefore, would be more present throughout the first trimester, there is evidence that miR-let-7d-3p regulates signaling pathways that change the neuronal differentiation cellular fate from neurogenesis to gliogenesis, and, for this reason, it appears with elevated expression mainly from the 16th week of pregnancy. (Graphical abstract).

Finally, it is important to emphasize the need for *in vitro* and *in vivo* studies. These types of studies could help clarify the reasons as to why miR-let-7d-3p and miR-451a are capable of influencing the mother-infant trajectory with direct effects in fetal neurodevelopment, or if these miRNAs would have an indirect action due to changes in diverse signaling pathways, culminating in lower or higher scores in cognitive and motor domains in 90 days babys. Also, studies are needed to clarify the mechanism of action of miR-let-7d-3p and miR-451a in the context of neurodevelopment throughout pregnancy. Additionally, longitudinal studies should be conducted to evaluate if the relationship between the miRNAs in question and the infant's cognitive and motor development remains throughout time, as neuroplasticity in this stage of life may correct differences in neurodevelopment, mitigating the effects observed in babies with more than three months of age. Lastly, larger population studies should be conducted to engage a larger sample size to corroborate our findings, as well as the analysis of other microRNAs in the peripheral blood of pregnant women that could be related to the motor and cognitive development in 90 days old infant's.

Therefore, this study showed that miR-let-7d-3p and miR-451a expression in the peripheral blood of pregnant women during the first/second trimester of pregnancy is associated with infant neuropsychomotor development at 90 days of life. What enlightens the perspective of using these microRNAs as biomarkers for infant neuropsychomotor development. Additionally, our articles showed that there could be a biological interrelation between the microRNAs miR-let-7d-3p and miR-451a when considering the cognitive domain of infants with 90 days postpartum. This brings now evidence for microRNA basic biology in the maternal-fetal axis.

## Author statement contributors

F. Nedel is the main author of the manuscript and responsible for the biological analyzes.

C. P. Ferrúa, C. C. do Amaral, G. P. Corrêa, A. B. Klug, R. G. Silveira and G. K. da Cunha participated in the acquisition of data and assisted in the biological analysis. A. P. Ardais, T. B. Fogaça, K. A. T. Pinheiro, R. K. S. S. Bast, G. Ghisleni, L. D. de M Souza, M. B. de Matos, L. de A. Quevedo and R. T. Pinheiro contributed to the conception and design of the study. J. Trettim and R. T. Pinheiro contributed to the statistical analyses and writing of the paper. R. T. Pinheiro is the project manager. All authors provided feedback on drafts of the manuscript and interpreted the results and all authors have approved the final manuscript.

## Research involving human participants

All subjects gave written informed consent for the analysis and anonymous publication of research findings. Those women at risk of suicide were referred to the public health service. This research was approved by the Research Ethics Committee of the Catholic University of Pelotas under the protocol 47807915.4.0000.5339.

## Funding and acknowledgments

This work was supported by Ministry of Health (DECIT) Brazil, CNPq/Brazil (Process 401726/2015-0 APP/Call 47/2014), Bill & Melinda Gates Foundation (INV-007186/OPP1142172) and INCT-EN (National Institute of Science and Technology, Brazil). Also we thank all pregnant women who participated in our study.

## Declaration of competing interest

The authors have declared no conflict of interest.

Ricardo Tavares Pinheiro.
