# Supplementary material for: Maternal expression of miR-let-7d-3p and miR-451a during gestation influences the neuropsychomotor development of 90 days old babies: "Pregnancy care, healthy baby" study
Source: J Psychiatr Res. 2023 Feb;158:185–91. doi: 10.1016/j.jpsychires.2022.12.021 (PMC9907453; doi:10.1016/j.jpsychires.2022.12.021)
Supplement: Multimedia component 1 [file mmc1.docx]

| **Supplementary table**: Data from the other variables used for adjustment by multiple linear regression. | | | | | | |
| --- | --- | --- | --- | --- | --- | --- |
|  | **Cognitive** |  | **Language** |  | **Motor** |  |
|  | **β (CI 95%)** | **p-value** | **β (CI 95%)** | **p-value** | **β (CI 95%)** | **p-value** |
| **Maternal age** (up to 23 years)* | -0.2 (-4.8;4.4) | 0.935 | -0.1 (-2.9;2.6) | 0.922 | 2.9 (-0.3;6.0) | 0.075 |
| **Socioeconomic level** (higher levels A/B)* | 1.4 (-3.8;6.5) | 0.607 | -2.0 (-4.4;0.5) | 0.115 | 0.7 (-3.6;4.9) | 0.762 |
| **Maternal schooling** (in years) | 0.5 (-0.3;1.3) | 0.162 | 0.2 (-0.5;0.7) | 0.660 | 0.2 (-0.4;0.8) | 0.547 |
| **Gestational age in the capture** (in weeks) | 0.3 (-0.1;0.7) | 0.168 | 0.0 (-0.3;0.3) | 0.865 | 0.4 (0.1;0.7) | 0.018 |
| **Maternal gestation body mass index** (weight/height^2^) | -0.4 (-0.8;0.1) | 0.056 | -0.2 (-0.4;0.2) | 0.285 | -0.4 (-0.7;-0.1) | 0.019 |
| **Multiparous** (no)* | -4.8 (-9.9;0.4) | 0.068 | -1.0 (-4.1;2.2) | 0.523 | -5.9 (-10.2;-1.6) | 0.008 |
| **Planned pregnancy** (yes) | -3.6 (-8.9;1.8) | 0.190 | 1.1 (-2.3;4.2) | 0.532 | 1.5 (-2.7;5.6) | 0.484 |
| **Degree of placental maturity** (degree I)* | -0.6 (-5.3;4.0) | 0.786 | -1.7 (-4.2;0.9) | 0.188 | -0.3 (-4.1;3.5) | 0.891 |
| **Prematurity** (no)* | -8.5 (-16.4;-0.4) | 0.040 | -6.7 (-11.5;-1.9) | 0.007 | -4.8 (-12.5;3.0) | 0.220 |
| **Type of birth** (vaginal)* | -3.1 (-8.2;1.9) | 0.220 | -1.4 (-4.6;1.8) | 0.370 | -5.5 (-9.5;-1.6) | 0.007 |
| **Weight of infant at birth** (in kg) | -1.9 (-8.1;4.4) | 0.555 | 0.9 (-3.0;5.0) | 0.638 | 5.5 (1.5;9.5) | 0.007 |
| **Baby sex** (male)* | -0.5 (-6.2;5.3) | 0.869 | -2.1 (-5.0;0.8) | 0.152 | 1.8 (-2.6;6.2) | 0.417 |
| **Postpartum depression** (no)* | 0.4 (-8.3;9.0) | 0.942 | -1.2 (-6.2;3.8) | 0.632 | -5.6 (-12.0;0.8) | 0.084 |
| **Breastfeeding** (no)* | 0.3 (-6.6;7.1) | 0.939 | 0.1 (-4.1;4.3) | 0.969 | 1.7 (-3.5;7.0) | 0.510 |
| **MDE** (control)* | 6.6 (1.7;11.5) | 0.009 | 2.3 (-0.5;5.2) | 0.106 | 6.6 (2.6;10.6) | 0.001 |
| *Reference category  CI=Confidence Interval |  |  |  |  |  |  |
